# Supplementary material for: Thioesterase induction by 2,3,7,8-tetrachlorodibenzo-p-dioxin results in a futile cycle that inhibits hepatic β-oxidation
Source: Sci Rep. 2021 Aug 3;11:15689. doi: 10.1038/s41598-021-95214-0 (PMC8333094; doi:10.1038/s41598-021-95214-0)

**Thioesterase induction by 2,3,7,8-tetrachlorodibenzo-*p*-dioxin results in a futile cycle**

**that inhibits hepatic β-oxidation**

Giovan N. Cholico^1,2^, Russell R. Fling^2,3^, Nicholas A. Zacharewski^1^, Kelly A. Fader^1,2^, Rance Nault^1,2^, Timothy R. Zacharewski^1,2,*^

^1^Biochemistry & Molecular Biology, Michigan State University, East Lansing, MI 48824, USA

^2^Institute for Integrative Toxicology, Michigan State University, East Lansing, MI 48824, USA

^3^Microbiology & Molecular Genetics, Michigan State University, East Lansing, MI 48824, USA

List of included materials: Supplementary Methods, Table S1, Figure S1, Figure S2, Figure S3

**SUPPLEMENRTARY METHODS**

**ECHS1 Enzymatic Activity Assay with TCDD**

Hepatic extracts were prepared from control mice treated with sesame oil vehicle every 4 days for 28 days. Total protein lysates were isolated from frozen samples with NP-40 cell lysis buffer (Thermo Fisher Scientific, Waltham, MA) with protease inhibitor using a Polytron PT2100 homogenizer (Kinematica). ECHS1 activity assays (n=3) were performed in 100 mM Tris buffer (pH 8.0) supplemented with 0.1 mg/ml bovine serum albumin at 37°C for 5 min. Reactions were started with 0.02 mg/ml total protein lysate, 0.025 mM crotonyl-CoA, and 10 or 100 nM TCDD (or 0.1% DMSO control). ECHS1 activity was measured by following the decrease in absorbance over time at 263 nm using a SpectraMax ABS Plus (Molecular Devices, San Jose, CA). Enzymatic activity was calculated using 6700 cm-1 M-1 as the extinction coefficient.

**Table S1.** **Effect of TCDD on β-oxidation intermediate levels.**

|  | **Compound ID** | **Description** | **C_n_** | **Score** | **Fold-Change (TCDD vs. Veh.)** | | | | |
| --- | --- | --- | --- | --- | --- | --- | --- | --- | --- |
|  |  |  |  |  | **0.3 µg/kg** | **1 µg/kg** | **3 µg/kg** | **10 µg/kg** | **30 µg/kg** |
| **Acyl-CoA** | HMDB59623 | Hexadecanoyl-CoA | 16 | 34.5 | **13.08** **± 3.06*** | 1.86 ± 0.33 | 6.98 ± 2.14 | 0.94 ± 0.78 | 0.00 ± 0.00 |
|  | HMDB01521 | Tetradecanoyl-CoA | 14 | ND |  |  |  |  |  |
|  | HMDB03571 | Dodecanoyl-CoA | 12 | ND |  |  |  |  |  |
|  | HMDB06404 | Decanoyl-CoA | 10 | 30.5 | **2.18** **± 0.42*** | 0.39 ± 0.06 | 0.57 ± 0.17 | 0.16 ± 0.16 | **0.01** **± 0.00*** |
|  | HMDB01070 | Octanoyl-CoA | 8 | 36.8 | 1.77 ± 0.63 | 0.04 ± 0.01 | 0.28 ± 0.04 | 0.06 ± 0.03 | 0.01 ± 0.01 |
|  | HMDB02845 | Hexanoyl-CoA | 6 | 37.2 | 1.43 ± 0.47 | **0.08 ± 0.02*** | 0.36 ± 0.05 | 0.08 ± 0.02 | **0.03 ± 0.02*** |
|  | HMDB01088 | Butyryl-CoA | 4 | 44.3 | 1.24 ± 0.33 | **0.17 ± 0.05*** | 0.57 ± 0.10 | **0.09 ± 0.01*** | **0.08 ± 0.04*** |
|  | HMDB01206 | Acetyl-CoA | 2 | 52.1 | 0.95 ± 0.12 | **0.30** **± 0.05*** | **0.53 ± 0.07*** | **0.04 ± 0.01*** | **0.16 ± 0.06*** |
| **Trans-2- Enoyl-CoA** | HMDB03945 | Hexadecenoyl-CoA | 16 | ND |  |  |  |  |  |
|  | HMDB03946 | Tetradecenoyl-CoA | 14 | ND |  |  |  |  |  |
|  | HMDB03712 | Dodecenoyl-CoA | 12 | ND |  |  |  |  |  |
|  | HMDB03948 | Decenoyl-CoA | 10 | ND |  |  |  |  |  |
|  | HMDB03949 | Octenoyl-CoA | 8 | 34 | 1.66 ± 0.19 | 1.14 ± 0.23 | 26.16 ± 25.31 | **114.31** **± 4.89*** | **139.82** **± 6.88*** |
|  | HMDB03944 | Hexenoyl-CoA | 6 | ND |  |  |  |  |  |
|  | HMDB62466 | Butenoyl-CoA | 4 | 35.1 | 1.05 ± 0.14 | **0.30** **± 0.05*** | **0.56** **± 0.07*** | **0.05** **± 0.03*** | **0.16** **± 0.07*** |
| **3-Hydroxy- Acyl-CoA** | HMDB62261 | Hydroxyhexadecanoyl-CoA | 16 | ND |  |  |  |  |  |
|  | HMDB03934 | Hydroxytetradecanoyl-CoA | 14 | ND |  |  |  |  |  |
|  | HMDB62260 | Hydroxydodecanoyl-CoA | 12 | ND |  |  |  |  |  |
|  | HMDB03938 | Hydroxydecanoyl-CoA | 10 | ND |  |  |  |  |  |
|  | HMDB03940 | Hydroxyoctanoyl-CoA | 8 | ND |  |  |  |  |  |
|  | HMDB03942 | Hydroxyhexanoyl-CoA | 6 | 35.3 | 0.86 ± 0.20 | **0.15** **± 0.03*** | **0.49** **± 0.07*** | **0.03** **± 0.01*** | **0.03** **± 0.01*** |
|  | HMDB01166 | Hydroxybutyryl-CoA | 4 | 51.3 | 0.82 ± 0.19 | **0.38** **± 0.13*** | **0.52** **± 0.14*** | **0.00** **± 0.00*** | **0.00** **± 0.00*** |
| **3-Keto- Acyl-CoA** | HMDB06402 | Oxohexadecanoyl-CoA | 16 | ND |  |  |  |  |  |
|  | HMDB03935 | Oxotetradecanoyl-CoA | 14 | ND |  |  |  |  |  |
|  | HMDB03937 | Oxododecanoyl-CoA | 12 | ND |  |  |  |  |  |
|  | HMDB03939 | Oxodecanoyl-CoA | 10 | ND |  |  |  |  |  |
|  | HMDB03941 | Oxooctanoyl-CoA | 8 | ND |  |  |  |  |  |
|  | HMDB03943 | Oxohexanoyl-CoA | 6 | ND |  |  |  |  |  |
|  | HMDB01484 | Acetoacetyl-CoA | 4 | ND |  |  |  |  |  |

β-Oxidation intermediate levels were assessed using untargeted liquid chromatography tandem mass spectrometry. Mice (n=4-5) were orally gavaged every 4 days for 28 days with sesame oil vehicle or TCDD. Fold-changes were calculated for each treatment group relative to the vehicle control group. Bold font and asterisks (*) denote a statistical significance (*p* ≤ 0.05) as determined using a one-way ANOVA with a Dunnett’s post-hoc analysis. Scores were determined using Progenesis by 60 being the maximum value and 0 being the minimum value. Scores ranging from 30 - 40 are based on mass error and isotope distribution similarity, while score >40 are based on mass error, isotope distribution and fragmentation score. All annotated compounds have a score distribution averaging ~35. Metabolites that were not detected are denoted with “ND”.

**
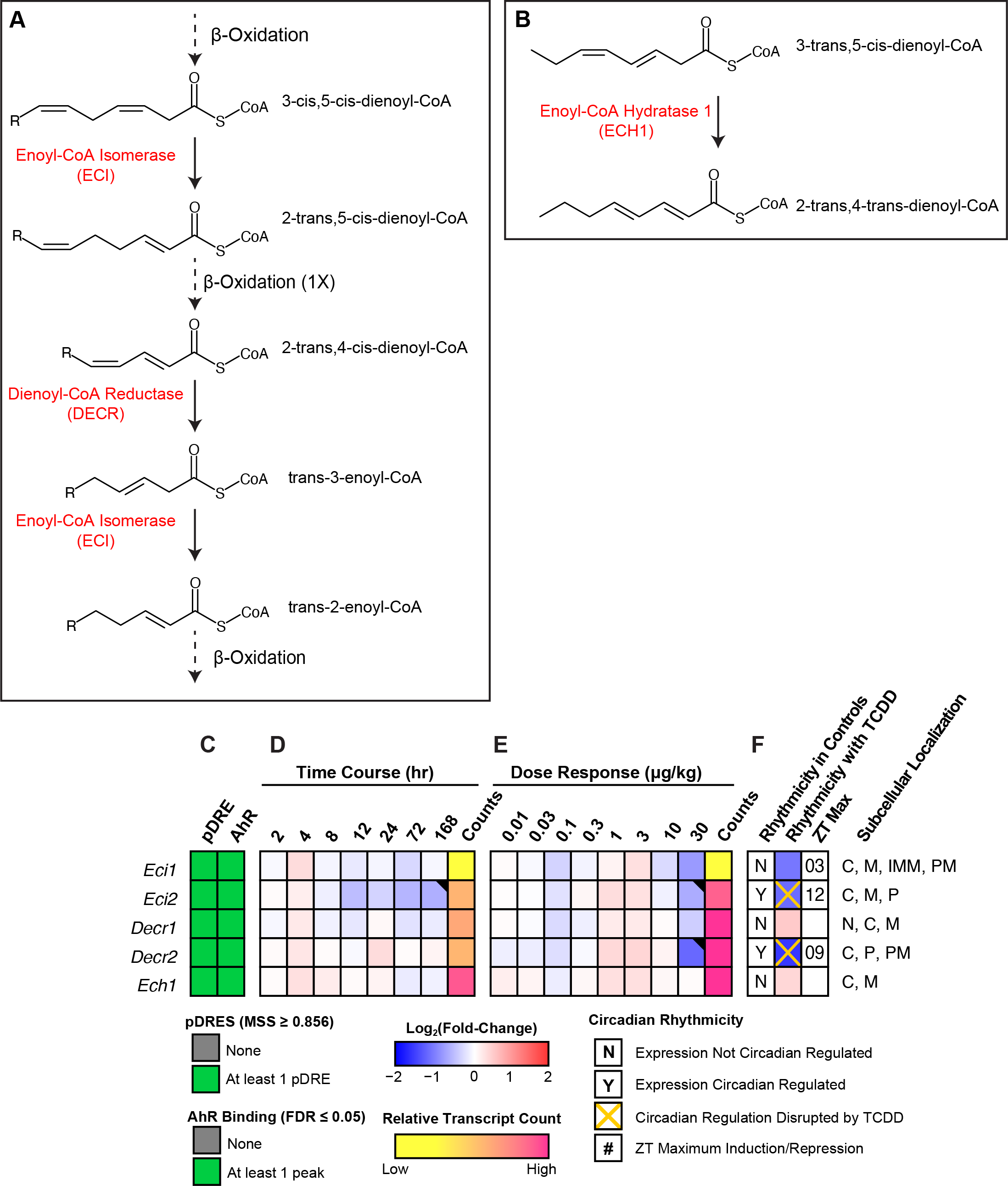
Figure S1.** **Effect of TCDD on the differential expression of auxiliary genes associated with unsaturated fatty acid oxidation.** **(A)** β-Oxidation of polyunsaturated fatty acids involves the use of the two auxiliary enzymes, enoyl-CoA isomerase (ECI) and dienoyl-CoA reductase (DECR). **(B)** β-Oxidation of 3-trans,5-cis-dienoyl-CoA species requires enoyl-CoA hydratase 1 (ECH1) isomerization activity. Official gene symbol designated in the MGI database are listed. Differential expression of genes associated with unsaturated fatty acid metabolism. **(C)** The presence of putative dioxin response elements (pDREs) and AHR enrichment at 2 hrs. **(D)** Time-dependent gene expression was assessed following a single bolus dose of 30 μg/kg TCDD (n=3). **(E)** Dose-dependent gene expression following oral gavaged every 4 days for 28 days with TCDD (n=3). **(F)** Circadian regulated genes are denoted with a “Y”. An orange ‘X’ indicates abolished diurnal rhythm following oral gavage with 30 μg/kg TCDD every 4 days for 28 days. The ZT with maximum gene induction/repression is provided. Counts represent the maximum number of raw aligned reads for any treatment group. Low counts (<500 reads) are denoted in yellow with high counts (>10,000) in pink. Differential expression with a posterior probability (P1(*t*)) >0.80 is indicated with a black triangle in the top right tile corner. Protein subcellular locations were obtained from COMPARTMENTS and abbreviated as: cytosol (C), mitochondrion (M), inner mitochondrial membrane (IMM), peroxisome (P), and plasma membrane (PM). The heatmap was created using R (v4.0.4). The biochemical reaction was created using Adobe Illustrator (v25.2).

**Figure S2.** **Effect of TCDD on ECHS1 enzymatic activity.** ECHS1 activity in control extracts was monitored by assessing the depletion of crotonyl-CoA in the presence of TCDD (or DMSO control). Crotonyl-CoA has an absorbance at 263 nm. The plot was created using GraphPad Prism (v8.4.3).

**A**

**i)** Representative data for vehicle (0 μg/kg TCDD):


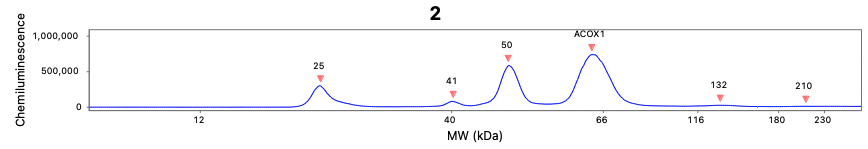


**ii)** Representative data for 1 μg/kg TCDD:


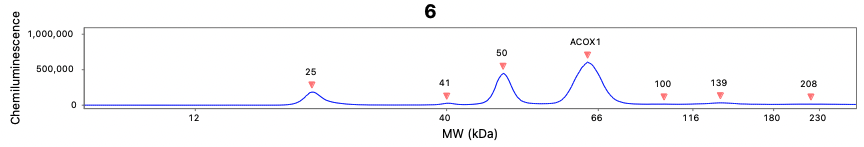


**iii)** Representative data for 3 μg/kg TCDD:


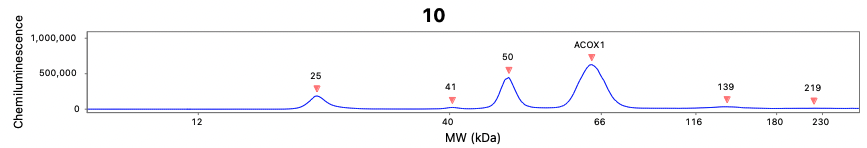


**iv)** Representative data for 10 μg/kg TCDD:


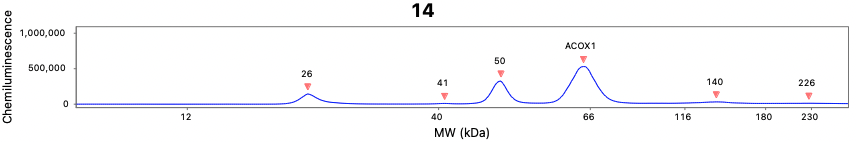


**v)** Representative data for 30 μg/kg TCDD:


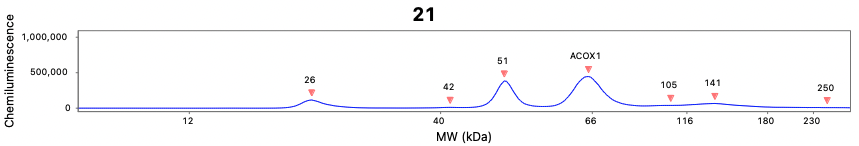


**Figure S3. Effect of TCDD on protein levels associated with β-oxidation.** Wes capillary electrophoresis was conducted to assess TCDD-induced changes in protein levels associated with β-oxidation. Representative spectra for vehicle (**i**), 1 μg/kg (**ii**), 3 μg/kg (**iii**), 10 μg/kg (**iv**), and 30 μg/kg (**v**) are depicted for ACOX1 (**A**), ACSL1 (**B**), ACSM3 (**C**), DBI (**D**), and ECHS1 (**E**). Spectra were created using Compass Software from ProteinSimple (v4.0.0). (*Continued on proceeding pages*)

**B**

**i)** Representative data for vehicle (0 μg/kg TCDD):


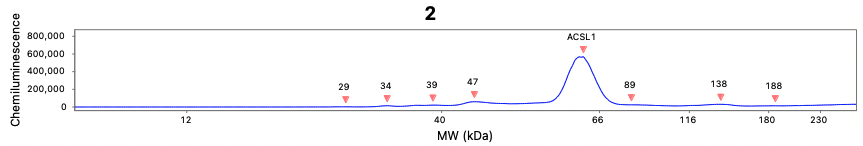


**ii)** Representative data for 1 μg/kg TCDD:


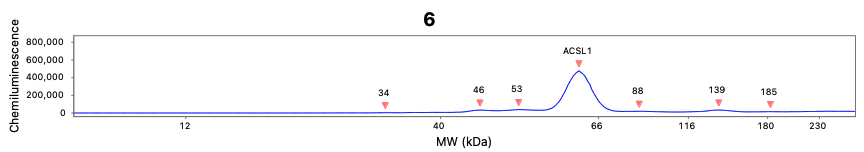


**iii)** Representative data for 3 μg/kg TCDD:


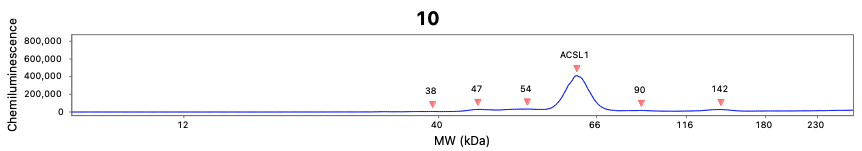


**iv)** Representative data for 10 μg/kg TCDD:


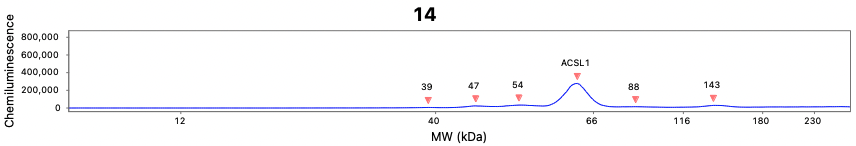


**v)** Representative data for 30 μg/kg TCDD:


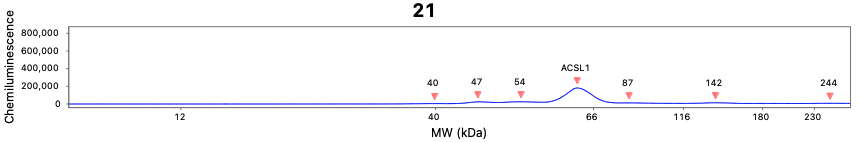


**C**

**i)** Representative data for vehicle (0 μg/kg TCDD):


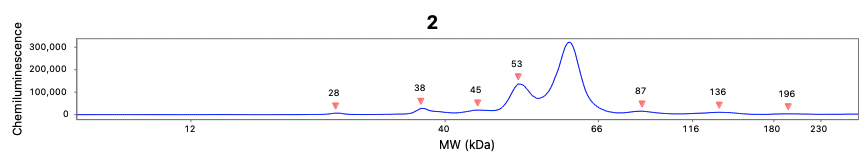


**ii)** Representative data for 1 μg/kg TCDD:


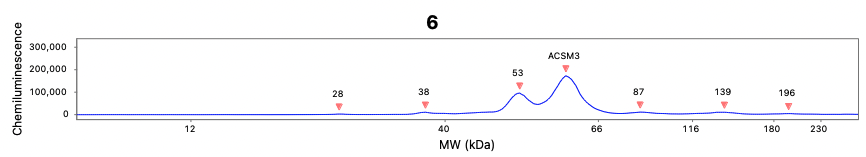


**iii)** Representative data for 3 μg/kg TCDD:


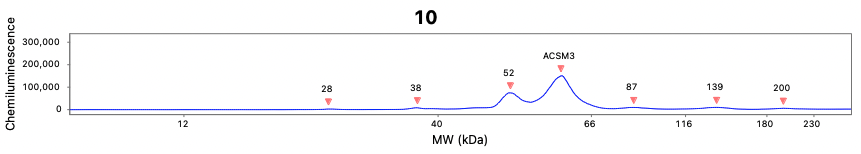


**iv)** Representative data for 10 μg/kg TCDD:


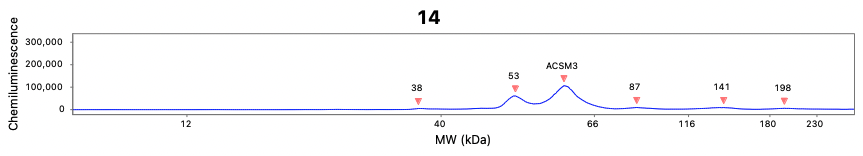


**v)** Representative data for 30 μg/kg TCDD:


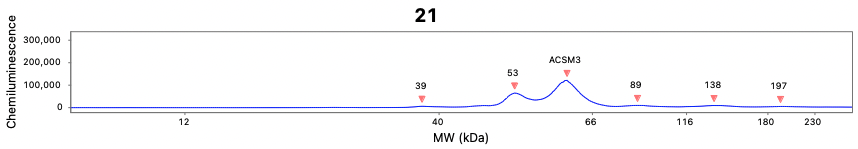


**D**

**i)** Representative data for vehicle (0 μg/kg TCDD):


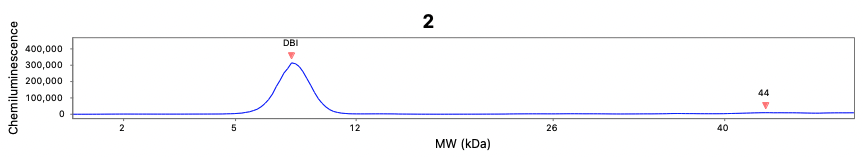


**ii)** Representative data for 1 μg/kg TCDD:


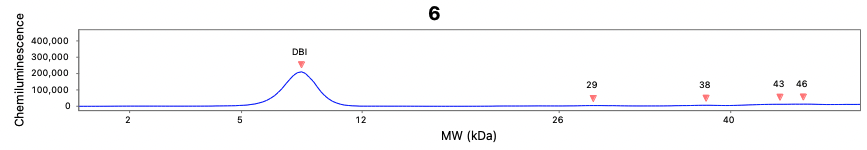


**iii)** Representative data for 3 μg/kg TCDD:


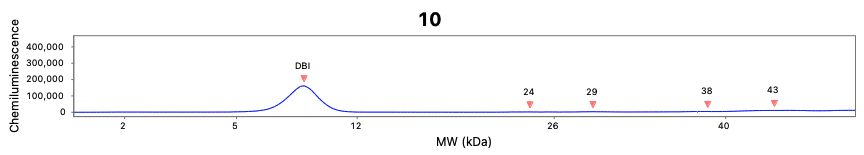


**iv)** Representative data for 10 μg/kg TCDD:


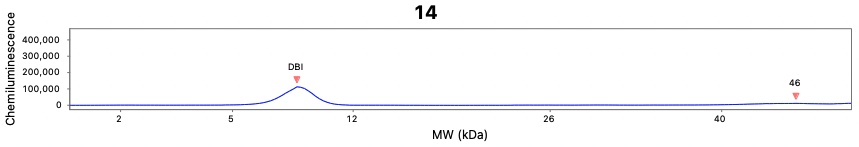


**v)** Representative data for 30 μg/kg TCDD:


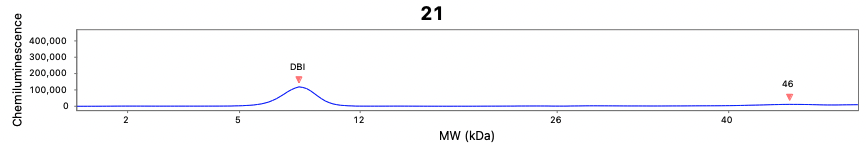


**E**

**i)** Representative data for vehicle (0 μg/kg TCDD):


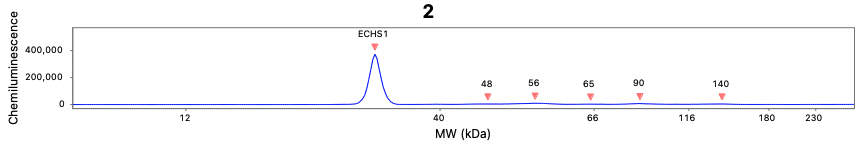


**ii)** Representative data for 1 μg/kg TCDD:


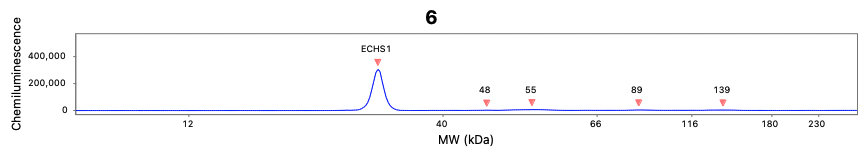


**iii)** Representative data 3 μg/kg TCDD:


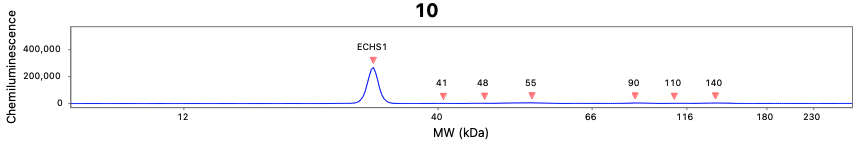


**iv)** Representative data for 10 μg/kg TCDD:


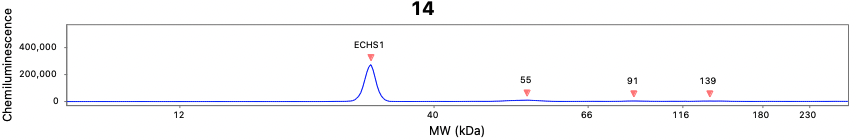


**v)** Representative data for 30 μg/kg TCDD:


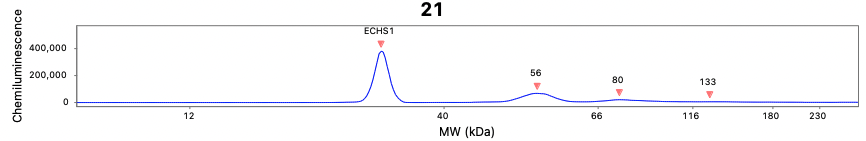

Supplement: Supplementary file 1 — Supplementary Information. [file 41598_2021_95214_MOESM1_ESM.docx]
